# The Relationship Between Cord Blood Cytokine Levels and Perinatal Characteristics and Bronchopulmonary Dysplasia: A Case–Control Study

**DOI:** 10.3389/fped.2022.807932

**Published:** 2022-04-07

**Authors:** Mengmeng Wang, Chenghan Luo, Zanyang Shi, Xinru Cheng, Mengyuan Lei, Wenjun Cao, Jingdi Zhang, Jian Ge, Min Song, Wenqian Ding, Yixia Zhang, Min Zhao, Qian Zhang

**Affiliations:** ^1^Neonatal Intensive Care Unit, The First Affiliated Hospital of Zhengzhou University, Zhengzhou, China; ^2^Clinical Treatment and Follow-Up Center for High-Risk Newborns of Henan Province, Zhengzhou, China; ^3^Key Laboratory for Prevention and Control of Developmental Disorders, Zhengzhou, China; ^4^Department of Orthopaedics, The First Affiliated Hospital of Zhengzhou University, Zhengzhou, China; ^5^Health Care Department, The First Affiliated Hospital of Zhengzhou University, Zhengzhou, China; ^6^Medical Record Management Section, The First Affiliated Hospital of Zhengzhou University, Zhengzhou, China

**Keywords:** cord blood, cytokines, maternal, perinatal factors, bronchopulmonary dysplasia

## Abstract

**Objective:**

To establish the association between serial levels of inflammatory cytokines in cord blood and perinatal characteristics and bronchopulmonary dysplasia (BPD) in preterm infants.

**Methods:**

147 premature infants with gestational age ≤32 weeks who were born and hospitalized in the First Affiliated Hospital of Zhengzhou University between July 2019 and August 2021 were enrolled in this retrospective case–control study. Multiple microsphere flow immunofluorescence was used to detect seven cytokines in cord blood collected within 24 h of birth. Demographics, delivery characteristics, maternal factors, neonatal characteristics, and clinical outcomes were collected for the two groups. An unconditional logistic regression model was used in this study to assess the clinical variables.

**Results:**

IL-6 cord blood levels at birth were significantly higher in the BPD group than in the non-BPD group, but the odds ratio (OR) was very small (OR = 1). No differences in other cytokine concentrations were observed between the two groups. Multivariable logistic regression analysis demonstrated that increased maternal white blood cell (WBC) count on admission and lower birth weight increased the risk of BPD progression.

**Conclusions:**

Increased IL-6 cord blood levels at birth in preterm infants may have trivial significance for predicting BPD. Furthermore, higher maternal WBC count on admission and lower birth weight increased the risk of BPD.

## Introduction

In 1967, Northway et al. first introduced the term bronchopulmonary dysplasia (BPD) to describe pulmonary injury in premature infants with respiratory distress syndrome (RDS) due to oxygen therapy and mechanical ventilation that showed imaging evidence of chest abnormalities at 36 weeks post-menstrual age (1). The prevalence of BPD has not decreased proportionately with advances in neonatal intensive care health care for respiratory management, including surfactant administration, perinatal steroids, and protective mechanical ventilation strategies, such as non-invasive positive pressure ventilation or high-frequency oscillatory ventilation, which have significantly improved the survival rate of preterm infants (2). BPD continues to be a common chronic respiratory disorder of preterm infants that affects ~20–40% of very-low-birthweight infants (3). BPD causes long-term pulmonary morbidities and places an enormous economic burden on society and families (4). BPD is correlated with an increased incidence of clinical sequelae during hospitalization and after discharge, prolonged hospital stay, recurrent respiratory infections, and adverse neurodevelopmental outcomes throughout infancy (5). Consequently, identifying a useful biological marker that predicts BPD in premature infants and conducting timely treatment intervention are essential to reduce pulmonary complications in premature infants with BPD.

Several studies have shown that perinatal, neonatal, and postpartum factors are related to the onset of BPD, and little is known about antenatal factors. Our research has shown that a high maternal white blood cell (WBC) count on admission increases the risk of BPD, which suggests that perinatal factors related to systemic inflammation predispose patients to the development of BPD. Many factors, including preterm birth, exposure to supplemental oxygen, mechanical ventilation, proinflammatory mediators with inflammation, perinatal infection, inherited genetic factors, and postnatal infection, are involved in the progression of BPD (6). Among these factors, pulmonary inflammation plays a crucial role in the pathologic process of BPD. Lung tissue inflammation is exacerbated by mechanical ventilation and oxygen exposure, and the release of proinflammatory cytokines into lung tissue during the inflammatory process further aggravates lung injury in preterm infants (7). Previous studies have revealed that proinflammatory cytokine concentrations in the serum and bronchoalveolar fluid lavage of preterm infants that were obtained within 24 h of birth are correlated with increased incidence of BPD (8). It is difficult to collect samples of amniotic fluid and tracheal aspirate from premature infants, whereas cord blood is easy to obtain for measurement of cytokine levels. In some studies, cytokine levels in umbilical cord samples obtained after birth were analyzed in relation to major neonatal complications, including BPD (9). Few studies have been performed thus far on the relationship of inflammatory cytokine concentrations in cord blood samples obtained at birth and maternal characteristics and BPD.

Accordingly, this study aims to analyze the value of cytokine concentrations from cord blood samples obtained within 24 h of birth as a non-invasive biomarker and to determine clinical variables for identifying independent risk factors for the occurrence of subsequent BPD in premature infants.

## Methods

### Study Design and Population

We performed a retrospective case–control study that was approved by the ethics committee of the First Affiliated Hospital of Zhengzhou University and for which informed parental consent was provided by all participants. The inclusion criteria were as follows: (1) a gestational age at birth below 32 weeks, (2) admission immediately after birth to our neonatal intensive care unit from July 2019 to August 2021 and survival until discharge, and (3) detection of cytokines within the first day of birth. The exclusion criteria were as follows: (1) major congenital malformations (e.g., congenital heart disease, multiple malformations, and chromosomal abnormalities), (2) incomplete data, and (3) death before discharge. Among 147 preterm infants with a gestational age of ≤ 32 weeks that were hospitalized in our institution during the study period, 60 developed BPD, corresponding to an incidence of 40.8%.

### Data Collection

Clinical data on demographics, delivery characteristics, and maternal and neonatal factors were retrospectively obtained from the hospital's electronic medical record system. The demographic and delivery characteristics included gestational age (GA), birth weight (BW), small for gestational age (SGA), sex, mode of delivery, placental abnormalities (including placenta previa, implantation and abruption), meconium-stained amniotic fluid, and 1- and 5-min Apgar scores. Data on maternal characteristics were also collected, including maternal age, multiple pregnancies, gestational hypertension, gestational diabetes mellitus (GDM), antenatal steroid administration, tocolytic treatment (e.g., progesterone), premature rupture of membranes (PROM), intrauterine growth restriction, embryo transfer, and maternal WBC count on admission. Neonatal characteristics and the presence of neonatal morbidities, including continuous positive airway pressure, mechanical ventilation, surfactant therapy, respiratory distress syndrome (RDS), intraventricular hemorrhage (IVH), patent ductus arteriosus (PDA), and sepsis, were obtained and recorded.

BPD was defined as a requirement for >21% supplemental oxygen at 28 postnatal days, which was consistent with the standards of the National Institutes of Health (10). RDS and IVH were diagnosed according to corresponding criteria (11, 12). PDA was identified as an infant needing indomethacin or ligation to close the ductus arteriosus. Echocardiography was the standard used to diagnose PDA (13). Infants with symptoms of sepsis and blood cultures showing bacterial growth were evaluated as having sepsis. Gestational age was estimated according to the last menstrual time and ultrasound diagnosis. A birth weight (BW) <10th percentile of the average weight for the respective gestational age was classified as small for gestational age (SGA) (14). PROM was defined as the occurrence of natural membrane rupture before delivery at a gestational age ≤ 37 weeks (15). Gestational hypertension was defined as a systolic blood pressure of 140 mmHg or more, a diastolic blood pressure of 90 mmHg or more, or both after 20 weeks of gestation (16). GDM was defined according to the guidelines of the American Diabetes Association (17).

### Cytokine Determinations

An early blood sample from the umbilical cord was collected within the first 24 h of life, and the concentrations of cytokines (IL-4, IL-6, IL-10, IL-12p70, IL-17, TNF-α, and IFN-γ) in the cord blood were detected by multiple microsphere flow immunofluorescence (using FACSCanto II flow cytometers produced by the BD Company, Qingdao, China). The cytokine detection reagent was provided by Qingdao Raisecare Biotechnology Co, Ltd., Qingdao, China (Lot Number: 2019801) according to the manufacturer's recommendations. The normal ranges of IL-4, IL-6, IL-10, IL-12p70, IL-17, and TNF-α were 0–8.56, 0–5.4, 0–12.9, 0–3.4, 0–21.4, 0–16.5, and 0–23.1 (pg/ml), respectively.

### Statistical Analysis

Unconditional logistic regression analysis was used to analyze clinical parameters significantly correlated to the onset of BPD. Quantitative data were obtained by performing a normality test (the Shapiro–Wilk test), and continuous variables were represented as by the mean ± SD if normally distributed and by medians and interquartile ranges otherwise. Qualitative data were analyzed by the χ^2^-test and reported as frequencies (*n*) and percentages (%). The data were analyzed using IBM SPSS Statistics v. 25 software. All analyses were performed using 2-sided tests, and *p* < 0.05 was regarded as statistically significant.

## Results

A total of 147 premature infants with gestational ages below 32 weeks were enrolled in our study, including 60 infants in a BPD group and 87 infants in a non-BPD group.

Based on demographic and delivery characteristics (Table 1), the birth weight [1,178.83 (278.77) vs. 1,332.76 (315.03), *p* = 0.004] of the BPD group was lower than that of infants without BPD. There were no significant differences in other clinical variables, including gestational age (GA), small for gestational age (SGA), sex, delivery mode, placental abnormalities, meconium-stained amniotic fluid, and 1- and 5-min Apgar scores.

**Table 1:** Demographic and delivery characteristics of the controls and infants with BPD.

	**BPD group**	**Control group**	**OR (95%CI)**	***P*** **-value**
	**(***n*** = 60)**	**(***n*** = 87)**		
Gestational age (wks), [median (IQR)]	29.30 (28.13, 30.20)	30.30 (29.10, 31.00)	0.939 (0.818, 1.078)	0.373
Birth weight (g), mean ± SD	1,178.83 ± 278.77	1,332.76 ± 315.03	0.998 (0.997, 0.999)	0.004*
SGA, *n* (%)	13 (21.7)	14 (16.1)	0.693 (0.300, 1.605)	0.392
Male sex, *n* (%)	26 (43.3)	37 (42.5)	0.968 (0.498,1.880)	0.923
Mode of delivery, cesarean, *n* (%)	44 (73.3)	53 (60.9)	0.567 (0.277, 1.160)	0.120
Placental abnormalities, *n* (%)	18 (30)	18 (20.7)	0.609 (0.285, 1.298)	0.199
Meconium-stained amniotic fluid, *n* (%)	14 (23.3)	21 (24.1)	1.045 (0.482, 2.267)	0.910
1 min Apgar score, [median (IQR)]	8 (6.25, 9)	8 (6.9)	0.965 (0.822, 1.134)	0.666
5 min Apgar score, [median (IQR)]	9 (8.10)	9 (8.10)	0.974 (0.781, 1.214)	0.816

*Values are expressed as mean ± SD, median (IQR), or n (%)*.

*IQR, Interquartile range; OR, odds ratio; CI, confidence intervals*.

*P-values were determined by univariate unconditional logistic regression analysis*.

* *Indicated statistical significance*.

The maternal perinatal data and clinical variables were comparable in both groups of infants as assessed by univariate analysis (Table 2). Mothers delivering preterm infants that developed BPD had a higher mean WBC count on admission than mothers delivering infants without BPD [11.74 × 10^9^/L vs. 10.23 × 10^9^/L; odds ratio (OR), 1.121; *p* = 0.02]. The difference remained insignificant in maternal age, multiple pregnancies, gestational hypertension, GDM, antenatal steroid administration, tocolytic treatment, PROM, intrauterine growth restriction, and embryo transfer rates between the two groups.

**Table 2:** Univariate regression analysis of perinatal factors of infants With BPD and controls.

	**BPD group**	**Control group**	**OR (95%CI)**	**P value**
	**(***n*** = 60)**	**(***n*** = 87)**		
Maternal age, [median (IQR)]	31 (28.35)	31 (29.34)	1.018 (0.954–1.085)	0.593
Multiple pregnancies, *n* (%)	13 (21.7)	26 (29.9)	1.541 (0.716–3.317)	0.269
Gestational hypertension, *n* (%)	21 (35)	29 (33.3)	0.929 (0.464–1.857)	0.834
Gestational diabetes mellitus, *n* (%)	12 (20)	20 (23)	1.194 (0.533–2.673)	0.666
Antenatal steroid, *n* (%)	8 (13.3)	15 (17.2)	1.354 (0.535–3.430)	0.523
Antenatal tocolytics, *n* (%)	25 (41.7)	23 (26.4)	0.503 (0.250, 1.014)	0.055
PROM, *n* (%)	18 (30)	25 (28.7)	0.941 (0.457–1.936)	0.868
IUGR, *n* (%)	5 (8.3)	5 (5.7)	0.671 (0.185–2.426)	0.543
Embryo transfer, *n* (%)	16 (26.7)	13 (14.9)	0.483 (0.212–1.099)	0.083
Maternal WBC count on admission (*10^9^/L), mean ± SD	11.74 ± 4.04	10.23 ± 3.29	1.121 (1.021–1.232)	0.017*

*Values are expressed as mean ± SD, median (IQR), or n (%)*.

*PROM, Premature rupture of membranes; IUGR, intrauterine growth restriction*.

*P-values were determined by univariate unconditional logistic regression analysis*.

* *Indicated statistical significance*.

Insignificant differences in both groups of infants in terms of neonatal characteristics and clinical outcomes could be detected, including main clinical therapeutic strategies, such as continuous positive airway pressure, mechanical ventilation, and surfactant therapy, as well as major clinical outcomes, including RDS, IVH, PDA, and sepsis (Table 3).

**Table 3:** Univariate regression analysis of neonatal characteristics and clinical outcomes of infants with BPD and controls.

	**BPD** **group** **(***n*** =6 0)**	**Control** **group** **(***n*** = 87)**	**OR (95%CI)**	***P*** **-value**
CPAP, *n* (%)	24 (40)	36 (41.4)	1.059 (0.542–2.069)	0.867
MV, *n* (%)	19 (31.7)	19 (21.8)	0.603 (0.286–1.270)	0.183
Surfactant use, *n* (%)	12 (20)	29 (33.3)	2.000 (0.922–4.336)	0.079
RDS, *n* (%)	57 (95)	82 (94.3)	0.863 (0.198–3.757)	0.845
IVH, *n* (%)	46 (76.7)	62 (71.3)	0.755 (0.354–1.610)	0.467
PDA, *n* (%)	14 (23.3)	21 (24.1)	1.045 (0.482–2.267)	0.910
Sepsis, *n* (%)	9 (15)	10 (11.5)	0.736 (0.280–1.937)	0.535

*Values are expressed as n (%)*.

*CPAP, continuous positive airway pressure; MV, mechanical ventilation; RDS, respiratory distress syndrome; IVH, intraventricular hemorrhage; PDA, patent ductus arteriosus*.

*P-values were determined by univariate unconditional logistic regression analysis*.

The IL-6 cord blood levels were significantly higher (median 102.69 vs. 25.07 pg/ml, *p* = 0.02) in the BPD group than in infants without BPD, but the OR was very small, and for every 1-pg/ml increase in the cord blood IL-6, there was a relatively small risk increase in the development of BPD [OR = 1; 95% confidence interval (CI), 1.000–1.000]. An insignificant difference was observed from cord blood samples in terms of other inflammatory cytokine concentrations (IL-4, IL-10, IL-12p70, IL-17, TNF-a, IFN-γ) between the BPD and non-BPD groups (Table 4).

**Table 4:** Univariate regression analysis of umbilical cord cytokine levels in infants with and without BPD.

	**BPD group** **(***n*** = 60)**	**Control group** **(***n*** = 87)**	**OR (95%CI)**	***P*** **-value**
IL-4, pg/mL	0.59 (0.30–1.30)	0.43 (0.22–1.16)	1.006 (0.989–1.023)	0.482
IL-6, pg/mL	102.69 (14.16–1,129.19)	25.07 (3.66–116.39)	1.000 (1.000–1.000)	0.016*
IL-10, pg/mL	2.43 (1.08–3.82)	2.01 (0.79–6.51)	0.997 (0.985–1.008)	0.569
IL-12p70, pg/mL	0.76 (0.29–1.24)	0.65 (0.23–1.40)	0.899 (0.701–1.153)	0.401
IL-17, pg/mL	0.77 (0.22–1.35)	0.53 (0.23–1.35)	0.915 (0.720–1.162)	0.466
TNF-a, pg/mL	0.60 (0.20–2.59)	1.20 (0.24–2.65)	0.901 (0.770–1.054)	0.192
IFN-γ, pg/ml	2.32 (1.03–4.89)	3.03 (1.19–8.21)	0.966 (0.913–1.021)	0.221

*Values are expressed as median (IQR)*.

*P-values were determined by univariate unconditional logistic regression analysis*.

* *Indicated statistical significance*.

The results of the aforementioned univariate analysis showed significant differences in the birth weight, maternal WBC count on admission, and IL-6 cord blood levels between the two groups of infants. All variables with a *p*-value < 0.05 were included in the multivariate logistic regression analysis, and the results demonstrated that an elevated maternal WBC count on admission (OR = 1.112; 95% CI, 1.006–1.229; *p* = 0.04) and low birth weight (OR = 0.998; 95% CI, 0.996–0.999; *p* = 0.003) were significantly associated with the development of BPD (Table 5).

**Table 5:** Multivariable logistic regression analysis showing the relationship between independent variables and the risk of BPD in infants with BPD and control groups.

	**OR**	**95%CI**	***P*** **-value**
Birth weight, g	0.998	0.996–0.999	0.003*
Maternal WBC count on admission, × 10^9^/L	1.112	1.006–1.229	0.037*
IL-6, pg/mL	1.000	1.000–1.000	0.010*

*P-values were determined by multivariable unconditional logistic regression analysis*.

* *Indicated statistical significance*.

## Discussion

It was demonstrated in this study that IL-6 cord blood levels within 24 h of birth were higher in the BPD group than in infants without BPD, and the difference was significant. An elevated maternal WBC count on admission was independently related to the occurrence of BPD. A multivariable regression analysis showed that this correlation remained after adjusting for other confounding factors, which has not been previously reported and suggests that the role of maternal perinatal inflammation may be critical for the pathologic process of BPD. In addition, low birth weight was independently associated with BPD occurrence. This result has been reported in previous studies (18).

IL-6 is a proinflammatory mediator that can induce lung lesions, aggravate long-term ventilation-induced barotrauma, and accelerate pulmonary inflammatory progression in the premature population, thus promoting pulmonary remodeling and the development of chronic lung disease (19, 20). IL-6 also increases inflammatory cytokine production (21). Elevated concentrations of the proinflammatory cytokine IL-6 have been demonstrated in both serum and tracheal aspirate (TA) samples collected on the first day of life of infants who subsequently developed BPD (22, 23). The results of our case–control study are more likely to demonstrate the true relationship between cytokine concentration profiles for cord blood obtained within 24 h of birth and BPD than previous reports. These findings indicate that among the investigated cytokines, cord blood values of IL-6 obtained within 24 h of birth are correlated with a higher incidence of BPD, which is consistent with previous findings (24). Although the *p*-value obtained from the multivariable logistic regression analysis showed a significant statistical difference in the IL-6 in cord blood obtained within 24 h of birth for the two groups, the OR was very small; thus, for every 1-pg/ml increase in cord blood IL-6, the impact on the increased risk of BPD was negligible, which suggests that IL-6 may have limited predictive value for the onset of BPD because the upper limit of 95% CI of the IL-6 level was close to 1.0.

At present, there is no relevant article on the relationship between umbilical cord blood IL-6 level within 24 h after birth and neonatal diseases. In our study, we found that the level of IL-6 cord blood at birth in the BPD group was significantly higher than that in the non-BPD group, but the OR was very small (OR = 1). This may be related to the late occurrence of BPD and the small gestational age of the cases we enrolled in the statistical analysis. In other words, the time span between the detection of umbilical cord blood IL-6 level within 24 h after birth and the occurrence of BPD is large, resulting in a smaller OR. In conclusion, to some extent, this suggests that cord blood IL-6 levels within 24 h after birth are associated with BPD.

This research demonstrated that maternal factors related to perinatal inflammation, such as an elevated maternal WBC count on admission, were correlated with BPD and have not been mentioned in previous literature, which suggest that the occurrence of BPD is associated with antenatal intrauterine inflammation to some extent. It is well known that an elevated WBC count indicates maternal infection. Maternal infections may have an impact on the state of neonates. Premature neonates are particularly vulnerable to infections because of immature immune defense systems, incompletely developed skin barriers, and frequent requirements for invasive operations (25). Fetal exposure to maternal infection has also been demonstrated to interrupt normal pulmonary vascular development and predispose preterm infants to the subsequent onset of BPD. This hypothesis is supported by a previous observation that perinatal maternal inflammation increases the expression of inflammatory factors in the fetal lung, which in turn affects the formation of alveoli and microvessels (26). However, in the case of maternal systemic infection, the exact pathogenesis of BPD is not completely understood. According to the current study and previous observation (27) demonstrating that proinflammatory cytokines scarcely pass through the placenta, maternal serum levels of IL-6, IL-8, TNF-α, and IFN-γ are not increased in preterm infants, suggesting that maternal inflammation status has limited predictive value for intrauterine infection and the development of BPD. Nevertheless, maternal exposure to inflammatory bacterial products can compromise neonates' innate immune systems, which has a tendency to exacerbate inflammation and infection. This finding provides further support for the hypothesis that maternal lipopolysaccharide administration might aggravate fetal inflammatory responses by triggering the production of reactive oxidative agents, leading to fetal proinflammatory cytokine expression (28). Proinflammatory mediators may contribute to the progression of BPD (29). The inflammatory response in the developing lung mediated by proinflammatory cytokines impacts normal alveolarization and impairs microvascular development (30).

The pathogenesis of BPD is multifactorial. However, a continuous inflammatory response may be a major contributor to the development of chronic lung disease (31). Cytokines are the principal regulators of intercellular communication and participate in mediating the inflammatory response, which are also involved in pulmonary vascular development, the mediation of acute lung lesions, and aggravate ventilator-induced lung injury (32, 33). High oxygen exposure appears to contribute to lung injury in premature infants, leading to the activation of proinflammatory cytokines (34). However, there is contradictory evidence demonstrating the cytoprotective role of IL-6 under exposure to hyperoxia (35). IL-6 can increase mortality, DNA damage, and apoptosis under exposure to hyperoxia and regulate angiogenesis in newborns (36). In summary, these observations indicate that the incidence of BPD may be multifactorial, and further investigation is needed to assess the function of IL-6 induced by hyperoxia exposure.

In previous studies, the IL-6 level in cord blood samples obtained at birth has been used as a predictor of the occurrence and progression of BPD in premature infants, and elevated IL-6 levels have been reported to suggest intrauterine infection (37). The rate of intrauterine infection is increased in PROM cases (38). Premature rupture of membranes is accompanied by an increase in the incidence of preterm birth (39). Premature infants have immature innate and adaptive immune responses that are characterized by insufficient synthesis of IgG, inadequate regulation and phagocytosis of pathogens, and higher activation of Th1 cells than Th2 cells, which may increase susceptibility to BPD (15). This indicates that the level of interleukin 6 in cord blood is related to the development of BPD.

Furthermore, cord blood samples can potentially be used as a non-invasive biological marker to predict the risk of major neonatal outcomes, including retinopathy of prematurity (40), RDS (41), necrotizing enterocolitis (42), sepsis (43), IVH (44), and hyperbilirubinemia (45). In this study, cord blood samples collected within 24 h of birth were used to predict the progression of BPD, and the results provide evidence of the predictive value of umbilical cord blood samples for neonatal diseases. However, cord blood cytokine concentrations may not reflect the lung cytokine concentration, as well as tracheal aspirate (TA) and amniotic fluid; therefore, an evaluation of human lung tissue is crucial to better ascertain the disease status (46). Further research is needed to explore this aspect.

In this study, low birth weight was implicated as an important factor for BPD. This result was in agreement with those of several studies (47). This result is also supported by previous reports that low-birth-weight infants are prone to develop BPD due to an immature antioxidative stress defense system resulting from exposure to hyperoxia at birth and a lack of surfactant (48). Insufficient surfactant can enhance susceptibility to RDS, thus increasing the incidence rate of BPD (49). Furthermore, studies have shown that birth weight is negatively correlated with the severity of BPD (50). This result further demonstrates that low birth weight is responsible for a high incidence of BPD.

This study has some potential limitations. First, the case–control design made it difficult to avoid selection bias and recall bias in the enrolled study population. Second, the relatively small sample led to low statistical efficiency of the research results, such that the reliability of the conclusion needs to be confirmed. Therefore, we cannot rule out the occasionality of a significant difference in cord blood interleukin 6 levels between the two groups of infants. Further studies on large samples are also needed to investigate an association between cord blood cytokine concentrations and the progression of BPD in premature infants. Limited by its retrospective nature, most of the data (such as maternal and other perinatal factors) used in this study were collected from an electronic medical record system before enrollment of subjects. Therefore, all the clinical indices were accurately recorded, and maternal pregnancy outcomes were assessed during data collection. The present study had several strengths. First, the researchers who collected samples, recorded clinical indexes, and measured cytokine levels were blind to each other, which reduced the possibility of selection bias. Second, as foreign populations were investigated in previous studies, the Chinese population investigated in our study fills a gap in this field.

## Conclusion

Our study demonstrates that inflammatory cytokine levels in cord blood obtained on the 1st day after birth may have limited predictive ability for the development of BPD. In addition, maternal characteristics associated with perinatal inflammation, such as elevated maternal WBC count on admission, have been found to be correlated with the onset of BPD. Therefore, further research on a larger number of enrolled infants is needed to investigate a potential association between measured concentrations of multiple cytokines in blood samples of premature infants and BPD. The role of interleukins as pulmonary biomarkers in the development of BPD and even timely therapeutic interventions during pregnancy also need to be investigated in the future.

## Data Availability Statement

The raw data supporting the conclusions of this article will be made available by the authors, without undue reservation.

## Author Contributions

QZ, MW, and CL designed the main purposes and methods of this study. MW wrote the article and QZ revised the article. ZS, XC, and ML participated in the index design and preliminary statistical analysis. WC, JZ, JG, MS, WD, YZ, and MZ participated in the data collection and critically reviewed the contents of the article. All authors agree to submit the final article and agree to be responsible for all aspects of the work.

## Funding

All phases of this study were supported by Science and Technology Department of Henan Province Project, No. 172102410017 (to QZ); Provincial and Ministerial Co-construction Project, No. SBGJ2018040 (to QZ); National Health Commission Medical and Health Science and Technology Development Center, No. VA2020HK41 (to QZ); Overseas Research and Training Project of Health Science and Technology Talents of Henan Province, No. HWYX2019066 (to QZ); Science and Technology Research Project of Henan Education Department, No. 20B320038 (to CL); and Joint Construction Project of Medical Science and Technology Public Relations in Henan Province, No. LHGJ20190064 (to ML).

## Conflict of Interest

The authors declare that the research was conducted in the absence of any commercial or financial relationships that could be construed as a potential conflict of interest.

## Publisher's Note

All claims expressed in this article are solely those of the authors and do not necessarily represent those of their affiliated organizations, or those of the publisher, the editors and the reviewers. Any product that may be evaluated in this article, or claim that may be made by its manufacturer, is not guaranteed or endorsed by the publisher.
